# Atomistic Simulation of Water Incorporation and Mobility in *Bombyx mori* Silk Fibroin

**DOI:** 10.1021/acsomega.1c05019

**Published:** 2021-12-15

**Authors:** Mathew John Haskew, Benjamin Deacon, Chin Weng Yong, John George Hardy, Samuel Thomas Murphy

**Affiliations:** † Department of Engineering, Lancaster University, Bailrigg, Lancaster LA1 4YW, U.K.; ‡ Department of Chemistry, Lancaster University, Bailrigg, Lancaster LA1 4YB, U.K.; § Scientific Computing Department, Science and Technology Facilities Council, Daresbury Laboratory, Warrington WA4 4AD, U.K.; ∥ Materials Science Institute, Lancaster University, Bailrigg, Lancaster LA1 4YB, U.K.

## Abstract

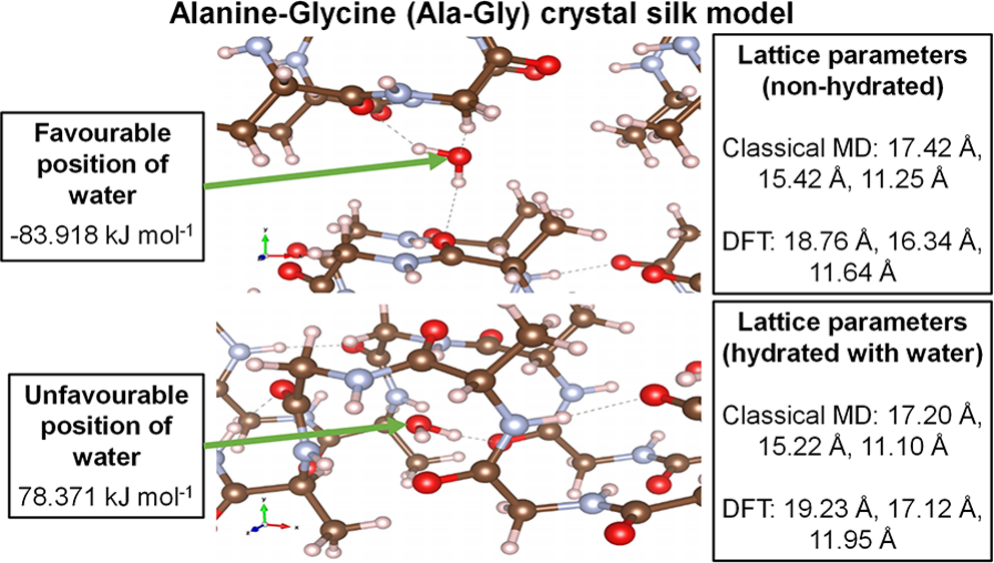

*Bombyx mori* silk fibroin (SF) is a biopolymer that can be processed into materials with attractive properties (e.g., biocompatibility and degradability) for use in a multitude of technical and medical applications (including textiles, sutures, drug delivery devices, tissue scaffolds, etc.). Utilizing the information from experimental and computational SF studies, a simplified SF model has been produced (alanine–glycine [Ala–Gly]_*n*_ crystal structure), enabling the application of both molecular dynamic and density functional theory techniques to offer a unique insight into SF-based materials. The secondary structure of the computational model has been evaluated using Ramachandran plots under different environments (e.g., different temperatures and ensembles). In addition, the mean square displacement of water incorporated into the SF model was investigated: the diffusion coefficients, activation energies, most and least favorable positions of water, and trajectory of water diffusion through the SF model are obtained. With further computational study and in combination with experimental data, the behavior/degradation of SF (and similar biomaterials) can be elucidated. Consequently, greater control of the aforementioned technologies may be achieved and positively affect their potential applications.

## 1 Introduction

Silk fibroin (SF) from the *Bombyx mori* silkworm is an Ala–Gly-rich protein, which is spun from aqueous solutions to produce strong and tough fibers.^1,2^ Furthermore, SF has excellent biocompatibility, making it a popular component of biomaterials.^3,4^ Many attempts have been made to mimic the natural process of producing robust silk filaments under mild environmental conditions.^5−8^ However, this has proven challenging, and many of the resultant fibers have been weaker than natural silk.^9^ Therefore, a greater understanding of the chemistry and properties of natural silk fibers (e.g., SF) is essential because this can help optimize the utilization of silk for various technical/medical applications.^10−16^

Natural silk fibers are semi-crystalline materials containing a mixture of secondary structures (e.g., β-sheets, helices, β-turns, and random coils) dependent on the species creating them.^17^*B. mori* SF can assume two distinct structures in the solid state,^1^ silk I and silk II (before and after spinning, respectively). Silk I is a β-turn type II conformation-rich structure, whereas silk II is an antiparallel β-sheet-rich structure.^1^ A common challenge when analyzing silk via X-ray diffraction or electron diffraction studies is the potential for the silk to convert from silk I to silk II.^17^ Other experimental techniques have also been applied, such as solid-state nuclear magnetic resonance, which is advantageous as the silk I form can be analyzed without reorientation or crystallization (and simultaneous conversion into silk II).^17^ Further details have been obtained using atomistic simulations on SF structures derived from NMR methods, such as 2D spin diffusion NMR, rotational echo double resonance, ^13^C chemical shift data, as well as X-ray diffraction data of a poly(Ala–Gly) sample.^1^

The *B. mori* SF macromolecule comprises three segments [heavy chain (HC) ca. 350 kDa, light chain (LC) ca. 26 kDa, and P25 ca. 25 kDa] in a ratio of 6:6:1.^18,19^ The HC is connected to the LC via a single disulfide link, while the P25 gene has non-covalent interactions with the HC and LC.^20^ Furthermore, the HC is made up of 5263 residues where glycine (Gly) is present in 45.9%, alanine (Ala) in 30.3%, serine (Ser) in 12.1%, tyrosine (Tyr) in 5.3%, valine (Val) in 1.8%, and 4.7% of the other amino acids.^21^ The HC possesses 12 repetitive domains that are Gly-rich, forming the crystalline regions, separated by short linker domains (42–43 residues). The short linker domains are non-repetitive and form amorphous regions.^17^ However, the repetitive domain is predominantly formed of Gly–X repeats (ca. 94% of the repetitive domain), where X is Ala (64%), Ser (22%), Tyr (10%), Val (3%), and threonine (Thr, 1.3%).^19,22^ The structural features of *B. mori* SF have been conveniently represented using the synthetic peptide, (Ala–Gly)_*n*_, where *n* is the number of repetitions of the Ala–Gly units, as a model for the crystalline regions.^18,23^ This is because the lack of Ser in the model peptide (Ala–Gly)_15_ does not affect the ^13^C cross-polarization magic angle spinning NMR chemical shifts of the Ala and Gly residues in the repeated sequence (Ala-Gly-Ser-Gly-Ala-Gly)_*n*_ of native SF.^17,24,25^ From X-ray and electron diffraction studies of *B. mori* SF, the periodic copolypeptide (Ala–Gly)_*n*_ has been shown to have an orthorhombic crystal structure with unit cell dimensions, *a* = 4.65 Å, *b* = 14.24 Å, and *c* = 8.88 Å, though these values are not the only reported unit cell dimensions for SF.^17,18,22,26^ Within the simplified (Ala–Gly)_*n*_ model, the repeat β-turn type II structure is stabilized by intramolecular hydrogen bond interactions. The overall planar sheets are held together by intermolecular hydrogen bonding interactions involving the central amide-bond of the β-turn, perpendicular to intramolecular interactions. Although such a (Ala–Gly)_*n*_ structural model vastly simplifies the overall structure of *B. mori* silkworm’s SF, it makes it less computationally demanding, thereby facilitating such studies.

Prior to spinning the *B. mori* SF fibers, the SF is stored in the middle silk gland (ca. 30% in water) and undergoes conformational changes when exposed to changes in the ionic composition of the spinning dope, mechanical stress, and loss of water during the natural fiber spinning process.^1^ The rate of degradation of SF is related to the content of the secondary β-sheet crystalline structure present within the bulk material.^28,29^ The β-sheet content from the regenerated SF can be modified through the use of various processing methods (e.g., water content and drying methods).^29,30^

Previously, molecular dynamic (MD) simulation was used to investigate the mechanical behavior of *B. mori* SF^27^ and the conformational change of its silk I form into silk II.^1^ The transformation of silk I into silk II is brought on by exposure to chemical/mechanical forces in an aqueous environment (i.e., the silk gland and spinneret).^1^ To simulate this structural change, a (Ala–Gly)_*n*_ model (at 298 K) was stretched [application of both shear (ca. 0.5 GPa) and tensile (ca. 0.1 GPa) stress] and the torsion angles of the residues evaluated. The resulting secondary structures showed a good agreement with existing solid-state NMR information indicating the potential of atomistic simulation techniques. The computationally produced silk II structure possessed ca. 75% β-sheet and ca. 25% β-turn content, comparable with experimental values of 73% β-sheet and 27% β-turn content.^1^

Despite extensive investigation of the *B. mori* SF structure discussed above, the fingerprint structural parameters for silk I and silk II remain mostly unexamined. Although the primary structure of *B. mori* SF contains a high content of (Ala–Gly)_*n*_,^26^ the SF can exist in either silk I or silk II form and its structural confirmations are less clear. It is generally accepted^31−33^ that silk II contains regions of orderly packed antiparallel β-sheets; however, the precise content varies between studies, which is in part caused by variations in experimental approaches/conditions and the variation of properties in natural materials.^34,35^ As for silk I, the structural parameters remain unclear because this conformation is less stable and susceptible to transformation into the silk II conformation, leading to difficulty in performing an analysis (e.g., X-ray diffraction experiments). As a result, multiple models exist for the silk I form (e.g., crankshaft model with Ala and Gly residues close to the β-sheet and α-helix confirmations,^25^ a loose fourfold helical confirmation,^36^ and a four-residue β-turn structure^37^). The possible structural models of *B. mori* SF (in silk I and silk II forms) have been examined using density functional theory (DFT) to determine the NMR chemical shifts. The DFT approach incorporated a similar (Ala–Gly)_*n*_ model mentioned previously and then calculated the ^13^C chemical shielding tensors using the theory-gauge independent atomic orbital with the Becke–Lee–Yang–Parr (BLYP) exchange–correlation functional.^38^ The results obtained indicated that the silk I structure did not entirely agree with that characterized by ^13^C NMR experiments. Instead, a 3_10_-helix-like conformation with torsion angle ranges of ⟨φ⟩ = −59 ± 2°, ⟨ψ⟩ = 119 ± 2° for the Ala residue and ⟨φ⟩ = −78 ± 2°, <ψ⟩ = 149 ± 2° for the Gly residue was suggested. However, the silk II structure agreed well with that characterized by ^13^C NMR experiments and previous descriptions of SF in the silk II form (i.e., the orderly packing of antiparallel β-sheets). The torsion angle ranges are ⟨φ⟩ = −143 ± 6°, ⟨ψ⟩ = 142 ± 5° for both Ala and Gly residues.^38^

As the utilization of SF expands, it is important to understand how the bulk material properties change when introduced to specific environmental conditions such as water. SF possess both hydrophobic and hydrophilic regions with a block copolymer design;^39^ therefore, solvents like water can easily interact with the SF protein structure. As a result, water molecules can cause a plasticizing effect, altering molecular interactions and impacting the mechanical properties of the material.^27,40^ In addition, a previous work has shown that when silk films are treated with methanol, they can exhibit an almost threefold increase in the β-sheet content when compared to water-annealed silk films.^41^ Consequently, it is important to understand how silk film material properties change with respect to this change in the β-sheet content. For instance, this information is important for the design of silk-based devices destined for in vivo applications (e.g., biocompatible and degradable batteries).^10^ Therefore, it is important to understand how the water content and organization of the SF secondary structure contribute to changes in the material.

Therefore, in this study, non-hydrated and hydrated SF crystal structure models were studied using both DFT and classical MD to understand how water is accommodated in the silk structures, what impact it has on the silk itself, and how it moves in the protein matrix to provide a unique insight into SF material properties. Utilizing DFT and MD techniques will highlight where water is orientated around the silk protein chains and the direction in which the water molecules diffuse throughout the structure and at various temperatures, particularly owing to the importance of water in the structure and degradation of silk-based materials used for various applications (e.g., SF utilized as a polymer electrolyte for energy storage devices^10^). Furthermore, this will facilitate future studies that investigate the incorporation of other molecules (e.g., charged ions such as Na^+^ or Mg^2+^) within the silk model, expanding the potential in how this material could be applied.

## 2 Results and Discussion

### 2.1 Lattice Parameters of (Ala–Gly)_*n*_ SF Crystal Models

Presented in Table 1 are the averaged lattice parameters for the silk structures equilibrated at 298 K for both the hydrated and non-hydrated simulation cells.

**Table 1:** Average Lattice Parameters for the Hydrated and Non-Hydrated Silk Structures at 298 K

	hydrated		non-hydrated	
parameter	DFT	classical MD	DFT	classical MD
*a*/Å	19.23	17.07	18.76	17.43
*b*/Å	17.12	15.11	16.34	15.43
*c*/Å	11.95	11.02	11.64	11.25

Table 1 shows that there is a significant discrepancy in the lattice parameters predicted by the DFT and classical MD simulations, with the DFT simulations predicting larger volumes. This discrepancy is likely a consequence of the subtle differences in the intermolecular and intramolecular forces, resulting in significant changes in the amount of folding of the Ala–Gly chains. Furthermore, there is an interesting difference in the two simulations techniques following the introduction of water. In the DFT case, hydrating the cell leads to an increase in the cell volume; however, in the classical MD, there is a slight decrease. This discrepancy indicates that there may be a significant difference in the silk–water interactions in the two models. Despite these differences, the impact on the water on the secondary structure appears to be similar for both techniques as described in the next section.

### 2.2 (Ala–Gly)_*n*_ SF Crystal Models’ Secondary Structure

**Figure 1:**
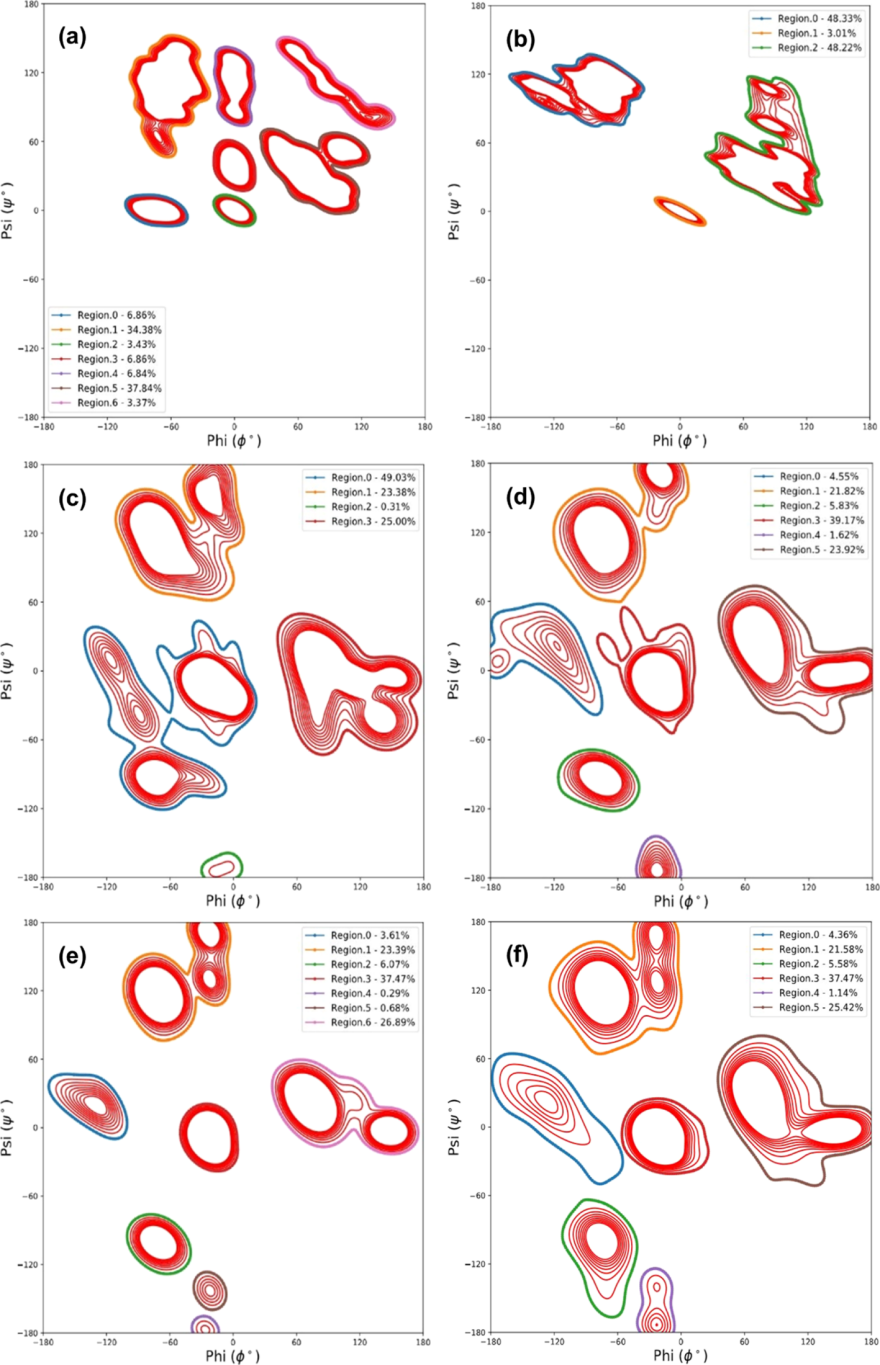
Ramachandran contour plot at 298 K of the DFT- and MD-generated torsion angles of the Ala and Gly residues from (Ala–Gly)_16_, (Ala–Gly)_128_, and (Ala–Gly)_1024_. The torsion angles of the residues from (Ala–Gly)_16_ were generated using DFT simulations, whereas the torsion angles of the residues from (Ala–Gly)_128_ and (Ala–Gly)_1024_ were generated using classical MD simulations. (a) Non-hydrated (Ala–Gly)_16_ SF crystal and the legend depicting the percentage of the residues (averaged over 154 fs) within each region. (b) Hydrated (Ala–Gly)_16_ SF crystal and the legend depicting the percentage of the residues (averaged over 69.7 fs) within each region. (c) Non-hydrated (Ala–Gly)_128_ SF crystal and the legend depicting the percentage of the residues (averaged over 2 ns) within each region. (d) Hydrated (Ala–Gly)_128_ SF crystal and the legend depicting the percentage of the residues (averaged over 2 ns) within each region. (e) Non-hydrated (Ala–Gly)_1024_ SF crystal and the legend depicting the percentage of the residues (averaged over 2 ns) within each region. (f) Hydrated (Ala–Gly)_1024_ SF crystal and the legend depicting the percentage of the residues (averaged over 2 ns) within each region.

Classical MD and DFT simulations were conducted to obtain the torsion angles for residues Ala and Gly. Figure 1 shows the Ramachandran contour plots of the non-hydrated and hydrated (Ala–Gly)_16_, (Ala–Gly)_128_, and the (Ala–Gly)_1024_ SF crystal models for clarity; only the 298 K NPT ensemble experiments are depicted. The torsion angles for (Ala–Gly)_128_ and (Ala–Gly)_1024_ were determined using classical MD simulations, whereas (Ala–Gly)_16_ utilized DFT simulations. With respect to the classical MD simulations for the Ramachandran contour plots, the 2 ns simulation time is sufficient given the constraints on the system (i.e., being more akin to a crystal). Figures S1 and S2 show the averaged residue positions of each system up to the first nanosecond and then up to the second nanosecond and shows that there is very little difference in the position of the residues and quantity of residues that occupy each space of the contour plot.

The inside of the contour plots appears empty, but this is not the case; the plateau of contour lines depicts a high number of residues within the regions (shown in Figure S3); as a result, the contour lines cannot be distinguished. Nevertheless, the Ramachandran plots in Figure 1 possess regions that lie within ⟨φ⟩ = −60° and ⟨ψ⟩ = 130° and ⟨φ⟩ = 70° and ⟨ψ⟩ = 10°, which is characteristic of the Ala and Gly residues, respectively, as reported in the literature.^1,67−70^ Here, it is indicated that the SF model utilized in this work possesses qualities that have been experimentally observed. Figure 1 indicates that the SF crystal models possesses a heterogeneous structure, evidenced by a left-handed α-helix, 3_10_-helix, β-sheet (ca. ⟨φ⟩ = 70° and ⟨ψ⟩ = 10°, ca. ⟨φ⟩ = −40° and ⟨ψ⟩ = −30°, ca. ⟨φ⟩ = −60° and ⟨ψ⟩ = 130°, respectively) and random coil structures. Furthermore, the (Ala–Gly)_*n*_ SF crystal structure can be considered to adopt the silk I form (i.e., repeated β-turn type II conformation) because β-sheets are not the predominant secondary structure; instead, the 3_10_-helix is the predominant secondary structure (ca. 37%), in agreement with the literature.^1,9,17,18,20,21,27,28,42,67−71^

On the other hand, by comparing the non-hydrated state of the (Ala–Gly)_*n*_ SF crystal model (Figure 1a,c,e) with the hydrated state (Figure 1b,d,f), the torsion angle regions for the Ala and Gly residues appear in slightly different locations. This is likely due to the flexibility of the SF model’s protein backbone (Ala and Gly) chains caused by the introduction of new hydrogen bond interactions. In addition, a lower number of hydrogen bond interactions between polymer chains within a system could hinder the reorganization of the system. As might be anticipated, as the temperature increases, the protein backbone chains can move more freely, resulting in a broadening of the Ala and Gly residues’ positions. Introduction of more hydrogen bond interactions from residues and/or water molecules (e.g., between protein backbone chains) impacts the potential flexibility of the (Ala–Gly)_*n*_ SF crystal model.

Moreover, the regions for the Ala and Gly residue positions for the non-hydrated and hydrated states of the larger (Ala–Gly)_1024_ SF crystal model (Figure 1e,f) are more defined compared to the (Ala–Gly)_128_ SF crystal model (Figure 1c,d). For instance, in Figure 1c, regions 0, 1, and 3 are distorted when compared to Figure 1e regions 1, 3, and 6. This could be due to a statistical difference between each system. (Ala–Gly)_1024_ possesses a greater number of dihedral angles; therefore, outliers are less likely to affect the overall residue region position/shape. In addition, the hydrated states in Figure 1d,f show many more similarities, which supports that the introduction of water molecules influences the positions of the Ala and Gly residues and their potential positions.

The torsion angles determined using DFT have also been included in Figure 1. These data further support the assertion that the SF crystal model possesses a heterogeneous structure. Therefore, they support the validity of the SF crystal model as the two different computational techniques predict similar secondary structures that also agree with the literature.^1,25,31−36,38,42^ Furthermore, a previous SF structure study using DFT chemical shift calculation (mentioned previously)^38^ reported that the torsion angle ranges for Ala and Gly are ⟨φ⟩ = −143 ± 6°, ⟨ψ⟩ = 142 ± 5°. In addition, the torsion angle range of ⟨φ⟩ = −143 ± 6°, ⟨ψ⟩ = 142 ± 5° is within the characteristic range for antiparallel β-sheets.^1,42^ In this work (Figure 1), the hydrated (Ala–Gly)_16_, (Ala–Gly)_128_, and (Ala–Gly)_1024_ cells have achieved similar torsion angle values for Ala and Gly residues.

### 2.3 Understanding the Behavior of Water in the (Ala–Gly)_*n*_ SF Crystal Models

**Figure 2:**
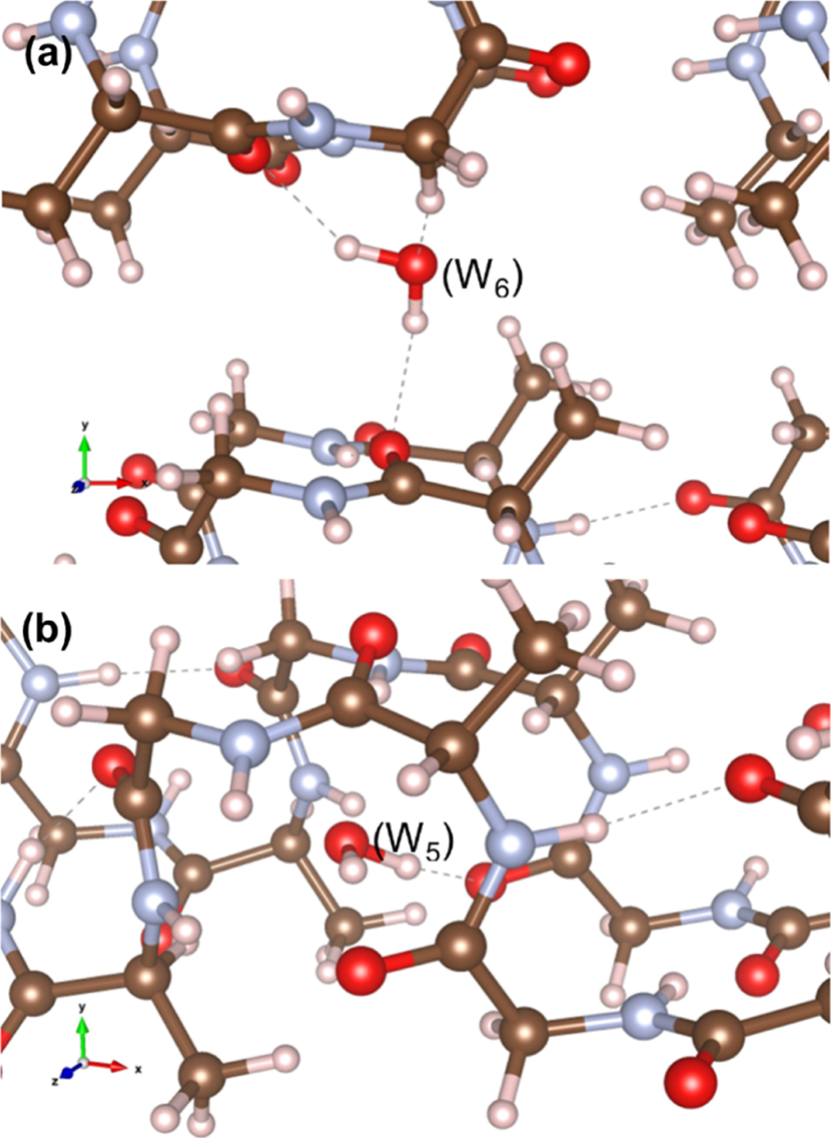
VESTA^44^ visualization of most (a) and least (b) favorable water molecule positions in the (Ala–Gly)_16_ SF crystal model (experimental data for SF crystal model from the literature^1,17,19,20,22,27,28^). The brown ball and sticks depict the carbon atoms; light gray, the hydrogen atoms; red, the oxygen atoms; and pale blue, the nitrogen atoms. The dashed lines depict the hydrogen bond interactions. In (a), the central water molecule (W_6_) is represented and in (b), the central water molecule (W_5_) is represented.

In order to understand the interactions between the water molecules and the polymer chains, the incorporation energy for water into a number of different positions in the simulation supercells was calculated using DFT.^72−74^ Locations for a single water molecule were selected randomly and the incorporation energy, *E*^inc^, calculated (eq 1).
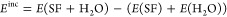
1where *E*(SF + H_2_O), *E*(SF), and *E*(H_2_O) are the energies of the energy minimized silk supercell containing the water molecule, the dehydrated silk structure, and the water molecule, respectively. The most favorable positions were those between polymer chains, enabling the formation of several hydrogen bonds as illustrated in Figure 2a. The incorporation energy for a water molecule incorporated as illustrated in Figure 2a is −83.92 kJ mol^–1^. The negative incorporation energy indicates that there is a thermodynamic driving force for water to collect between the chains. By contrast, water molecules found between the type II β turns (see Figure 2b) are found to have positive formation energies indicating that these are not favorable sites for incorporation of water.

**Figure 3:**
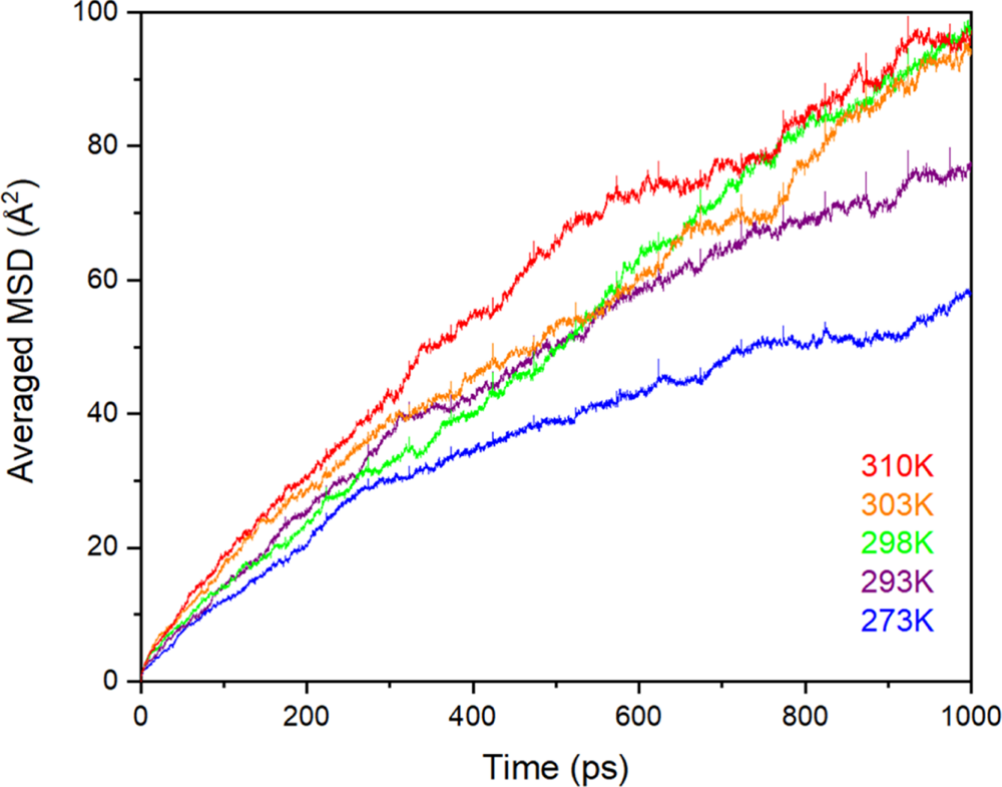
MSD of the oxygen ions from the water molecules (in Å^2^) in the hydrated SF crystal model (Ala–Gly)_1024_. The legend depicts the temperature (in K).

Having investigated possible locations for the accommodation of water molecules within the structure, we now examine the mobility of the water molecules around the silk. In order to obtain sufficient water diffusion to provide adequate statistics, the mean square displacement (MSD) calculations were performed using classical MD simulations of the largest 4 × 4 × 4 simulation supercells. The MSDs for the oxygen ions in the water molecule are reported in Figure 3.

**Figure 4:**
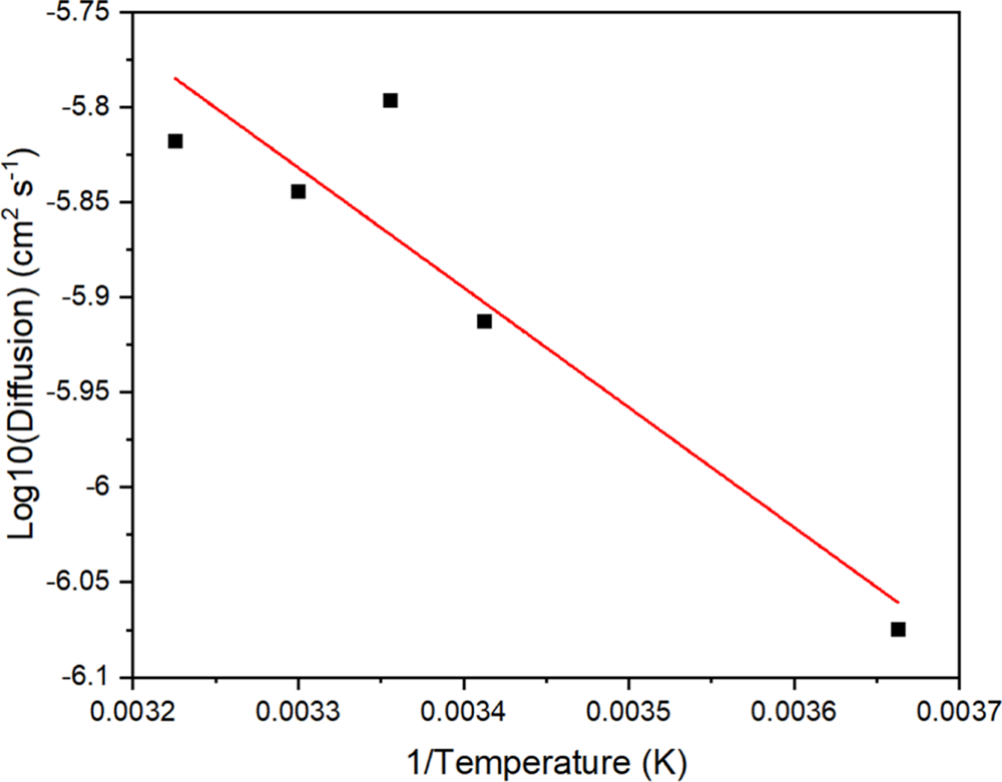
Arrhenius^75^ plot of the hydrated (Ala–Gly)_1024_ SF crystal at the temperature range of 273–310 K. By using the gradient of the slope in the Arrhenius plot, the calculated activation energy for water diffusion in the SF crystal is determined as 12.07 kJ mol^–1^.

Figure 3 shows that as the temperature increases, the MSD for the oxygen ion in the water molecule increases linearly with time, indicating that the water molecules are free to diffuse around the polymer chains, even at very modest temperatures. By taking the gradient of the MSDs and plotting against 1/temperature, it is possible to create an Arrhenius^75^ plot, as shown in Figure 4.

From the Arrhenius^75^ plot, it is clear that the data can be fitted using a straight line, yielding an activation energy of 12.07 kJ mol^–1^ and a maximal diffusion coefficient (*D*_0_) of 1.78 × 10^–4^ cm^2^ s^–1^.^76^ Moreover, a previous study on SF as an edible coating for perishable food preservation reported that their experimentally obtained diffusion coefficient (*D*), at room temperature, was 1.05 × 10^–6^ cm^2^ s^–1^ at a 58% β-sheet content, 3.21 × 10^–6^ cm^2^ s^–1^ at a 48% β-sheet content, and 5.79 × 10^–6^ cm^2^ s^–1^ at a 36% β-sheet content.^77^ Looking at room temperature (298 K), the diffusion coefficient predicted from Figure 4 is 1.60 × 10^–6^ cm^2^ s^–1^, which is similar to the experimental results. As mentioned above, the experimental samples had β-sheet contents greater than 36%, while the sample studied here has a lower β-sheet content (ca. 26%) that does appear to have an impact on the diffusivity.

**Figure 5:**
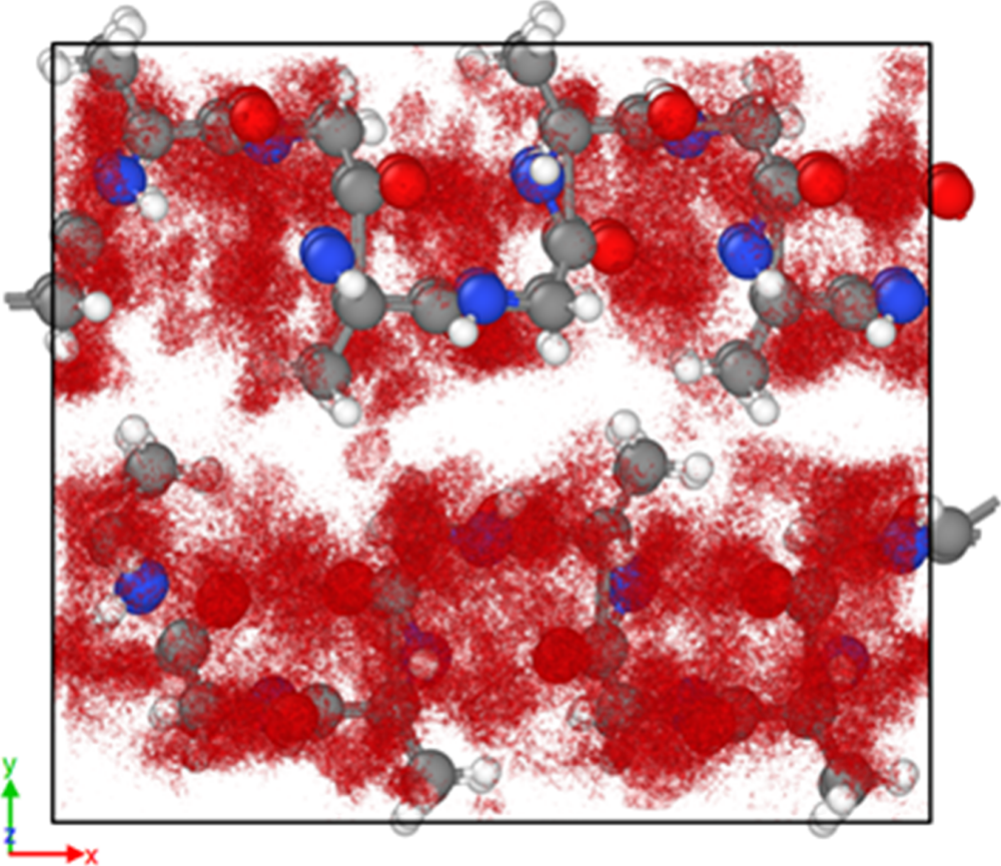
VESTA^44^ visualization of the water trajectory of the hydrated (Ala–Gly)_1024_ SF crystal at 298 K over 1 ns (experimental data for SF crystal model from the literature^1,17,19,20,22,27,28^). The dark gray ball and sticks depict the carbon atoms; white, the hydrogen atoms; red, the oxygen atoms; and blue, the nitrogen atoms. For the water molecules, only their oxygen atoms are used to depict their locations and the red isosurface represents the water density around the silk chains. In addition, the water density displayed represents the initial and final frame, whereas the Ala–Gly protein chain depicts only the final frame position of the residues.

**Figure 6:**
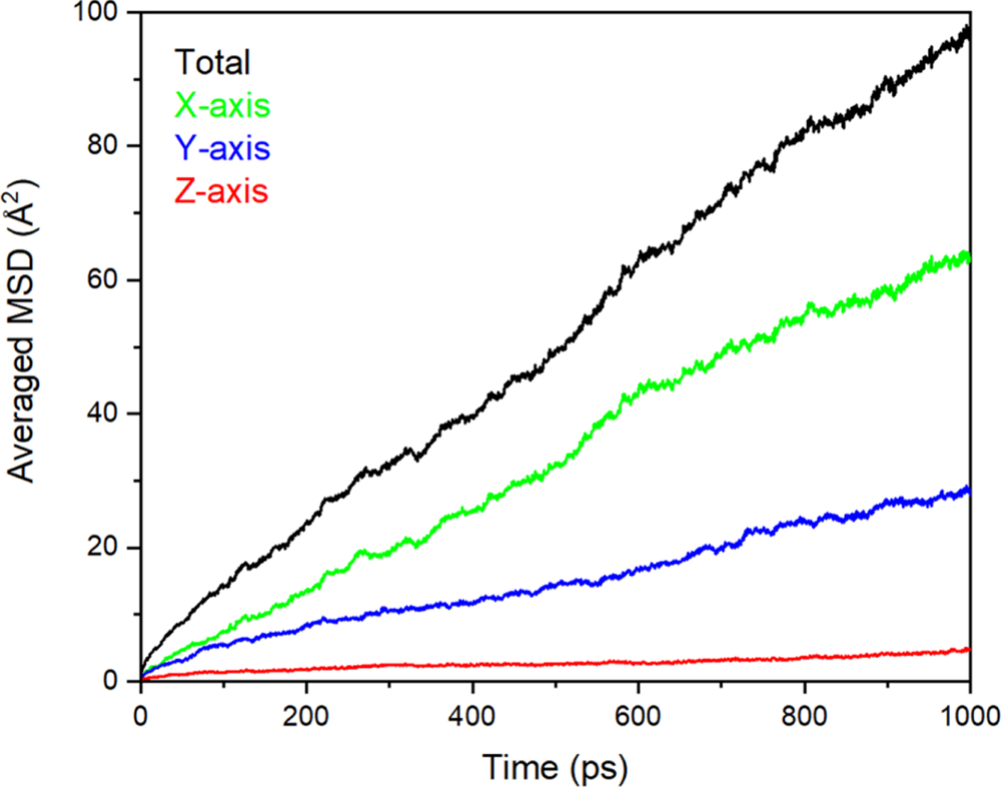
MSD of the oxygen ions from the water molecules (in Å^2^) in the hydrated SF crystal model (Ala–Gly)_1024_ at 298 K. The total MSD of the oxygen ions from the water molecules is represented by the black line, the MSD of the oxygen ions from the water molecules along the *X*-axis is represented by the green line, that along the *Y*-axis is represented by the blue line, and that along the *Z*-axis is represented by the red line.

Finally, we explore the dynamic motions of the water molecules to elucidate the diffusion mechanism around the polymer chains. Shown in Figure 5 are the trajectories of the water molecules around the silk from the classical MD simulations at 298 K over 1 ns. In addition, depicted in Figure 6 is the directional MSD of the oxygen ions from the water molecules (in Å^2^) in the hydrated SF crystal model (Ala–Gly)_1024_ at 298 K over 1 ns. From the trajectories, it is suggested that there is increased diffusion in the *X*-axis direction as there is little movement of water across the chains in the *Y*-axis direction, therefore indicating a high degree of anisotropy,^78^ as the water molecules tend to diffuse through the free space between the chains rather than crossing the chains. A similar pattern was also observed from the DFT simulations (supported by Figure 2); as a result, both the classical MD and the DFT simulations suggest that the diffusion of water around the silk protein chains will be anisotropic.

Figure 2a depicts that the most favorable position for water molecules to be within the SF crystal model is located across the Ala–Gly protein chains, as determined using DFT. As seen from Figure 5, numerous water molecules populate the areas across the protein chains; however, the water molecules mostly diffuse through the spaces between the protein chains (along the *X*-axis direction, as shown in Figure 6), therefore highlighting an agreement between our methods (classical MD and DFT) and potential for water incorporated into each SF system. Despite the differences in lattice parameters (Table 1), we do not observe a significant impact on the SF crystal’s secondary structure. Both MD and DFT methods produced similar results, first represented in Figure 1, which are also in good agreement with the literature.

## 3 Conclusions

The utilization of classical MD and DFT has provided a unique insight into SF-based biomaterials. The secondary structure was evaluated by comparing it to information available in the literature and demonstrating the efficacy of the simulation models that predicted the characteristic torsion angles for residues Ala and Gly. The DFT simulations provided insights into the β-sheet region residues, while the MD simulations enabled calculation of the percentages of the residues detailing the predominant secondary structures. In addition, the (Ala–Gly)_*n*_ SF crystals hydrated with water were investigated, and the displacement of water, diffusion coefficient, activation energy, energy of water positions, and trajectory were reported. As a result, an appreciation for combining different techniques to investigate the materials is obtained.

The information reported could be expanded upon for future work (e.g., introduction of a different solvent to the system and mechanical stress evaluation). With continued investigation into materials such as SF, we believe a greater understanding of their properties and key aspects/interactions can be achieved, positively impacting the applications of materials produced using SF and silk-inspired polymers and proteins.^79,80^ It is noted that these results correlate well with NMR studies of supercontracted spider silks showing that water is more able to permeate the amorphous matrix and not the semi-crystalline β-sheet-rich matrix^81^ and a degree of anisotropy in the motion and organization of water in the silks including in the hydration sphere of specific structural elements of the silks or reservoirs/voids that may play a role in supercontraction.^82,83^

## 4 Methodology

Utilizing both DFT and classical MD to study the structure of SF provides a description across a range of time and length scales. While DFT simulations can provide a detailed description of the electronic structure of the silk, the number of atoms accessible is insufficient to accurately assess the secondary structure of the protein chains.

By contrast, classical MD employs an empirical force field that is fitted to reproduce the intra- and intermolecular interactions between atoms/ions in the system. The form of the interaction potentials is based on considerations of the electronic structure; however, the parameterization remains fixed during the simulation and therefore, they are unable to represent processes, such as charge transfer and bond breaking and formation. On the other hand, this loss of electronic flexibility allows the simulation of thousands of atoms over long timescales, enabling analysis of the secondary structure and the examination of bulk water transport around the protein chains.

### 4.1 Preparation of the (Ala–Gly)_*n*_ SF Unit Cell

**Figure 7:**
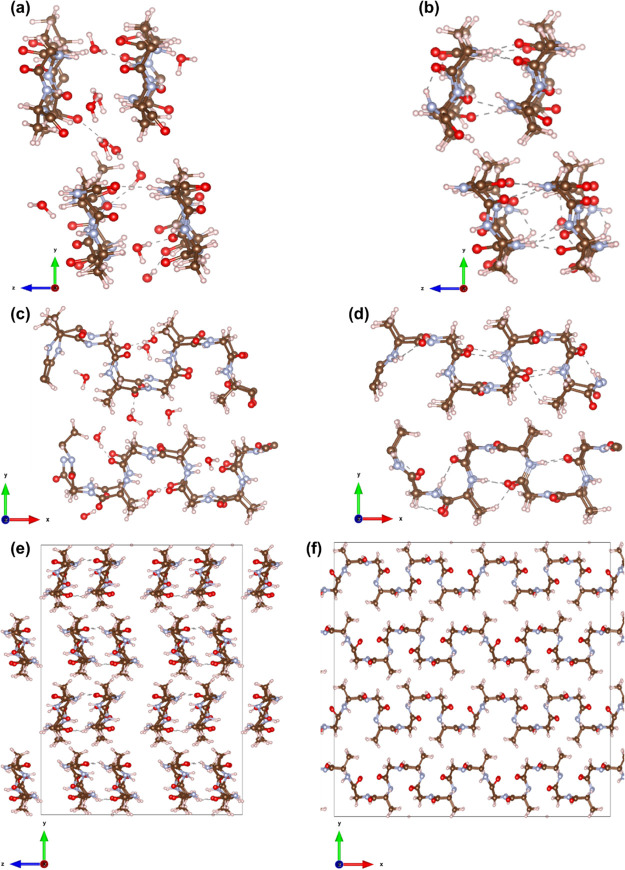
Visualization of the crystal structure of *B. mori* SF in a silk I form. (a) Snapshot of the hydrated crystal structure (repeated β-turn type II conformation) from along the *X*-axis, (b) non-hydrated from along the *X*-axis, (c) hydrated from along the *Z*-axis, and (d) non-hydrated from along the *Z*-axis. (e) Snapshot of a non-hydrated 2 × 2 × 2 periodic unit cell of a *B. mori* SF crystal structure from along the *X*-axis and (f) non-hydrated 2 × 2 × 2 periodic unit cell of *B. mori* SF crystal structure from along the *Z*-axis (experimental data for the SF crystal model from the literature^1,17,19,20,22,27,28^). A visual representation of the repeated β-turn type II conformation where the brown ball and sticks depict carbon atoms; light gray, the hydrogen atoms; red, the oxygen atoms; and pale blue, the nitrogen atoms. The initial lattice parameters of the unit cell shown in Figure 7 are orthorhombic: *a* = 17.8 Å, *b* = 15.7558 Å, *c* = 11.4904 Å. The gray dashed lines represent the hydrogen bond interactions. The figure was created using VESTA.^44^

Construction of the (Ala–Gly)_*n*_ SF unit cell followed the methodology described by Yamane et al.^1^ The initial unit cell was created by arranging four Ala–Gly chains with repeated β-turns according to information from the experiment and simulation on SF.^1,17,19,20,22,27,28^ To simulate the bulk system of the repeated polymer chains, a periodic boundary condition was implemented, where nitrogen and carbon-terminals were connected to mirror images of themselves, and the resulting structure is illustrated in Figure 7.

Hydrated SF supercells were created by introducing ca. 7.5 wt % of water molecules, mimicking experimentally reproduced SF films.^42,43^ Water molecules were placed randomly within the supercells while ensuring that no water molecules were placed within 1.7 Å of the silk.

### 4.2 Classical MD Simulations

Classical MD simulations utilized a range of supercells, ranging from 2 × 2 × 2 to 4 × 4 × 4 repetitions of the unit cell containing 2176 and 17,408 atoms (2377 and 19,022 atoms when hydrated). In each case, the DL_FIELD package was used to create the input files for the DL_POLY_4 simulation package.^45−48^ In addition, the periodic (Ala–Gly)_*n*_ crystals were visualized using the Visual Molecular Dynamics programme.^49^

Interactions between the ions in the (Ala–Gly)_*n*_ chains were represented using the all-atom optimized potentials for liquid simulations^50^ force field where the total energy (*E*_tot_) of a molecular system is evaluated as a sum of the following components, the non-bonded energy (*E*_nb_), bond stretching and angle bending terms (*E*_bond_ and *E*_angle_, respectively), and the torsional energy (*E*_torsion_).^45,51,52^ To represent interactions between water molecules and the silk chains, the three-site transferrable intermolecular potential force field was employed.^53,54^

The MD simulations were carried out by using the DL_POLY and DL_FIELD^47^ to construct the force field models and the necessary input files for DL_POLY.^46^ The van der Waals and Coulombic real space cut-off were set to 12 Å. The Coulombic interactions were treated by means of smooth particle mesh Ewald.^55^

During the equilibration and sampling processes in canonical (*NVT*) and isothermal–isobaric (*NPT*) ensembles, all the temperatures and pressures were maintained by using the Nose–Hoover formalism with the coupling constants set to 0.05 and 0.1 ps, respectively, at an atmospheric pressure of 1. A fixed timestep of 0.5 fs was used to update the trajectories.

The initial system configurations (both the non-hydrated and the hydrated ones) were optimized at a low temperature of 10 K at NVE for 50 ps. After that, the system was equilibrated in *NVT* at a target temperature of 10, 150, 273, 298, 310, 373, and 473 K, with each successive starting configuration obtained from the previously equilibrated configurations at the lower temperatures. These independent systems each with a target temperature as mentioned above were equilibrated for 100 ps, and then the system ensembles were changed to the NPT and the systems were equilibrated for a further 100 ps at each temperature, mentioned previously. Afterward, the sampling runs were taken, and the atomic configurations were written to the trajectory files every 1000 steps (0.5 ps) for a total of 2 ns. Dihedral angles were then calculated using the DL_ANALYSER^48^ package, enabling the creation of Ramachandran plots. Using the torsion angles of the Ala and Gly residues (water molecules are excluded), Ramachandran plots were prepared for the purpose of comparison with Ramachandran plots of SF/Ala and Gly.^56−58^

In order to examine the mobility of water around the SF, the MSD of the oxygen ions in the water molecule was determined. The simulation for the MSD of water molecules within the hydrated supercells was conducted in a similar manner as previously stated; however, the simulation progressed for a total of 1 ns. The MSD at time *t* is defined as an ensemble average (eq 2).
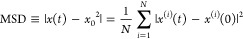
2where *N* is the number of particles to be averaged, vector *x*^(*i*)^(0) = *x*_0_^(*i*)^ is the reference position of the *i*th particle, and vector *x*^(*i*)^(*t*) is the position of the *i*th particle at time *t*.^59^

### 4.3 DFT Simulations

DFT simulations^60^ were carried out using the Quickstep method in CP2K,^61^ with the BLYP exchange correlation functional.^62,63^ A double zeta valence polarized (DZVP) basis set was employed for all calculations.^64^ The DZVP has been shown to perform well with single point and geometry optimization calculations, providing accurate results while also being computationally efficient.^65^ Due to the greater computational requirements, the DFT simulations were performed on supercells smaller than 2 × 2 × 2.

Non-hydrated and hydrated silk supercells were minimized until the forces on the atoms were below 10^–3^ eV/A. Simulation supercells were then equilibrated using ab initio molecular dynamics (AIMD) under *NPT* conditions at the same temperatures studied using classical MD. AIMD simulations used the Nose–Hoover thermostat and default CP2K barostat^66^ with a relaxation time of 60 fs for both and proceeded for 1500 fs, with a timestep of 0.5 fs. For sampling the non-hydrated and hydrated supercells, the final trajectory of the geometry optimized *NPT* ensemble simulations was used for the starting point. The non-hydrated supercell simulation proceeded for 3080 frames with each frame being 0.05 fs, while the hydrated supercell simulation proceeded for 1394 frames. This results in a total simulation time of 154 and 69.7 fs for the non-hydrated and hydrated supercells, respectively. The discrepancies in the length of the simulations were due to the available ARCHER cost.

Lastly, the trajectories of 8 water molecules (ca. 7.5% water content) through the hydrated periodic (Ala–Gly)_16_ crystal were also obtained from AIMD.^61^ This was possible by running MD simulations using the information obtained from the DFT-optimized hydrated periodic (Ala–Gly)_16_ crystal. However, the *NVT* ensemble was set (after the cell underwent equilibration for 1500 fs) for 310 K and a timestep of 0.05 fs. A total of 42,350 MD steps were carried out, which gave a total simulation time of 2117.5 fs.

## Supporting Information Available

Ramachandran contour plot at 298 K of the MD generated torsion angles of the Ala and Gly residues from the non-hydrated and hydrated (Ala–Gly)_128_; Ramachandran contour plot at 298 K of the MD generated torsion angles of the Ala and Gly residues from the non-hydrated and hydrated (Ala–Gly)_1024_; and Ramachandran plot at 298 K of the MD generated torsion angles of the Ala and Gly residues from (Ala–Gly)_128_ and (Ala–Gly)_1024_ (PDF)
