# Supplementary material for: Atomistic Simulation of Water Incorporation and Mobility in Bombyx mori Silk Fibroin
Source: ACS Omega. 2021 Dec 15;6(51):35494–504. doi: 10.1021/acsomega.1c05019 (PMC8717555; doi:10.1021/acsomega.1c05019)
Supplement: Supplementary file 1 — ao1c05019_si_001.pdf [file ao1c05019_si_001.pdf]

Supporting Information

Atomistic simulation of water incorporation and mobility in Bombyx mori silk fibroin

Mathew John Haskew[a,b], Benjamin Deacon[a], Chin Weng Yong[c], John George Hardy[b,d,*] and Samuel Thomas Murphy[a,d,*]

[a] Department of Engineering, Lancaster University, Bailrigg, Lancaster, LA1 4YW, UK.

[b] Department of Chemistry, Lancaster University. Bailrigg, Lancaster LA1 4YB, UK.

[c] Scientific Computing Department, Science and Technology Facilities Council, Daresbury Laboratory, Warrington, WA4 4AD, UK.

[d] Materials Science Institute, Lancaster University. Bailrigg, Lancaster LA1 4YB, UK.

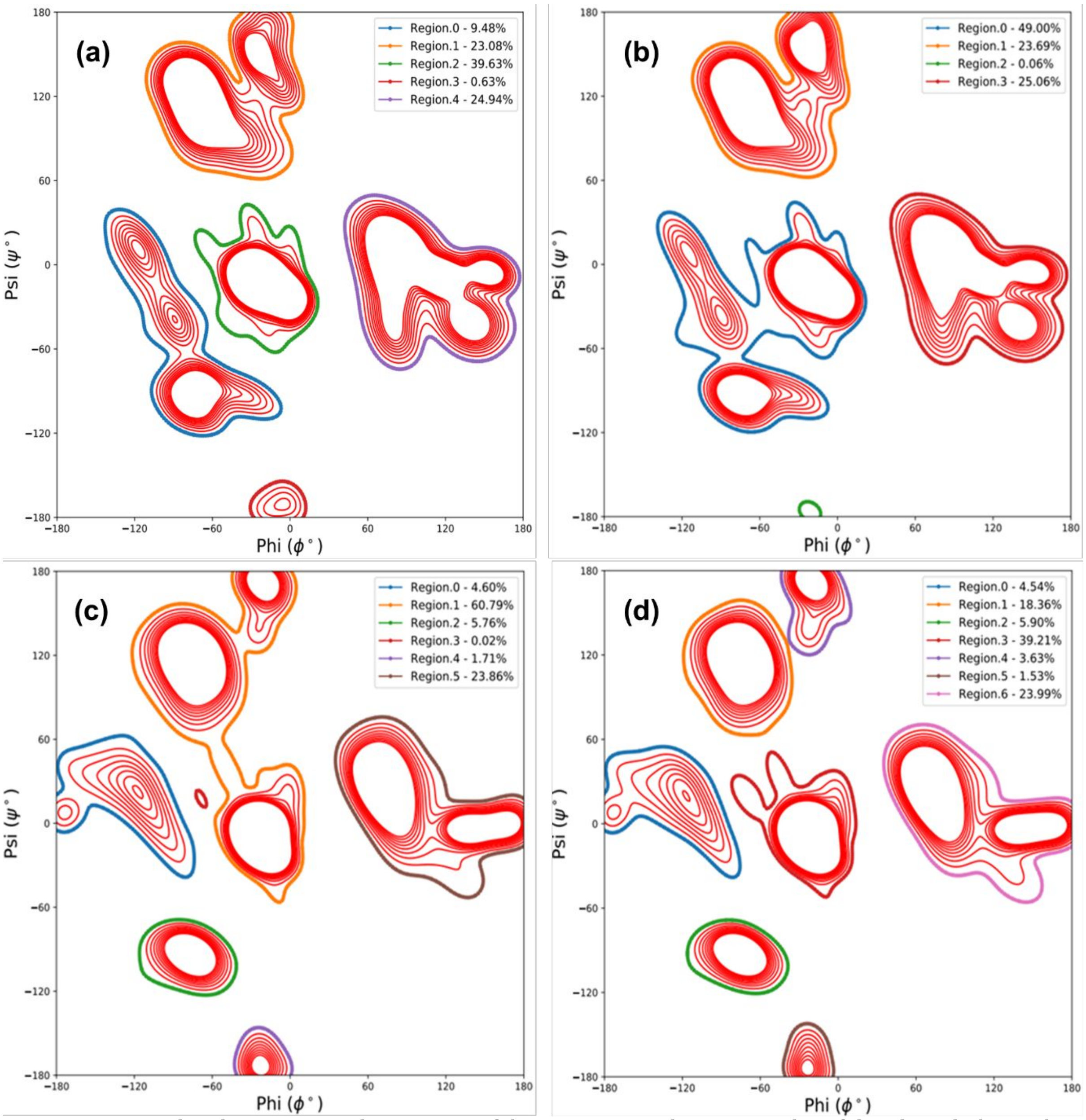

Figure S1. Ramachandran contour plot at 298 K of the MD generated torsion angles of the Ala and Gly residues from the non-hydrated and hydrated $(Ala\text{-}Gly)_{128}$. (a) Is the position of the residues (averaged over 0.5 to 1000 ps) in the non-hydrated system and the legend depicts the percentage of the residues within each region. (b) Is the position of the residues (averaged over 1000.5 to 2000 ps) in the non-hydrated system and the legend depicts the percentage of the residues within each region. (c) Is the position of the residues (averaged over 0.5 to 1000 ps) in the hydrated system and the legend depicts the percentage of the residues within each region. (d) Is the position of the residues (averaged over 1000.5 to 2000 ps) in the hydrated system and the legend depicts the percentage of the residues within each region.

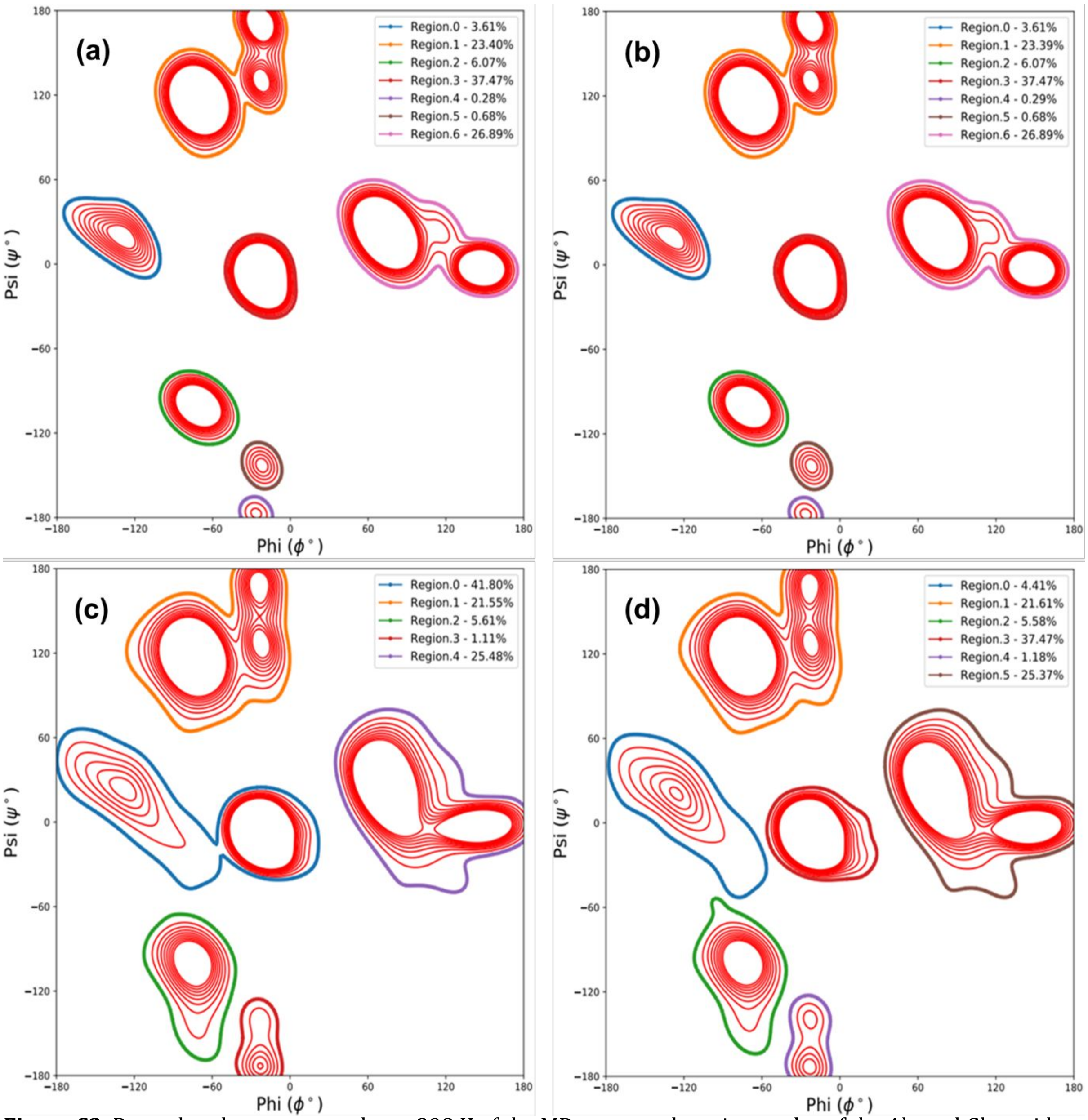

Figure S2. Ramachandran contour plot at 298 K of the MD generated torsion angles of the Ala and Gly residues from the non-hydrated and hydrated $(Ala\text{-}Gly)_{1024}$. (a) Is the position of the residues (averaged over 0.5 to 1000 ps) in the non-hydrated system and the legend depicts the percentage of the residues within each region. (b) Is the position of the residues (averaged over 1000.5 to 2000 ps) in the non-hydrated system and the legend depicts the percentage of the residues within each region. (c) Is the position of the residues (averaged over 0.5 to 1000 ps) in the hydrated system and the legend depicts the percentage of the residues within each region. (d) Is the position of the residues (averaged over 1000.5 to 2000 ps) in the hydrated system and the legend depicts the percentage of the residues within each region.

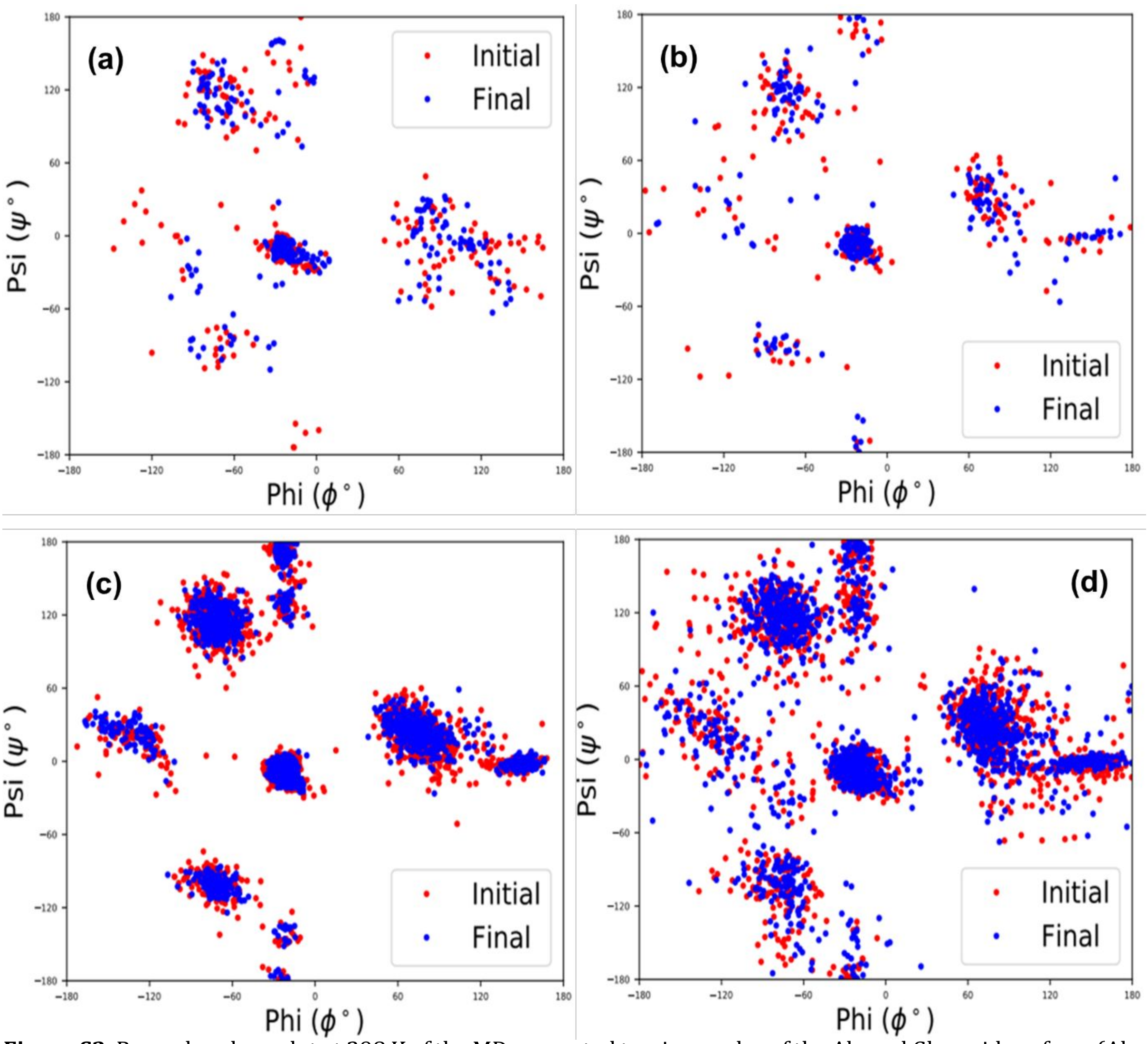

Figure S3. Ramachandran plot at 298 K of the MD generated torsion angles of the Ala and Gly residues from (Ala-Gly)$_{128}$ and (Ala-Gly)$_{1024}$. (a) Is the non-hydrated (Ala-Gly)$_{128}$ SF crystal and the legend depicts the initial (red dots) and final (blue dots) positions of the Ala and Gly residues at 2 ns sampling. (b) Is the hydrated (Ala-Gly)$_{128}$ SF crystal and the legend depicts the initial (red dots) and final (blue dots) positions of the Ala and Gly residues at 2 ns sampling. (c) Is the non-hydrated (Ala-Gly)$_{1024}$ SF crystal and the legend depicts the initial (red dots) and final (blue dots) positions of the Ala and Gly residues at 2 ns sampling. (d) Is the hydrated (Ala-Gly)$_{1024}$ SF crystal and the legend depicts the initial (red dots) and final (blue dots) positions of the Ala and Gly residues at 2 ns sampling.
